# Supplementary material for: Development and Regulation of the Extreme Biofilm Formation of Deinococcus radiodurans R1 under Extreme Environmental Conditions
Source: Int J Mol Sci. 2023 Dec 28;25(1):421. doi: 10.3390/ijms25010421 (PMC10778927; doi:10.3390/ijms25010421)
Supplement: Supplementary file 1 [file ijms-25-00421-s001.zip › ijms-2734587-supplementary.pdf]

Development and Regulation of the Extreme Biofilm Formation of *Deinococcus radiodurans* R1 under Extreme Environmental Conditions

Qiannan Guo [1,2,†], Yuhua Zhan [1,2,†], Wei Zhang [1,2], Jin Wang [1,2], Yongliang Yan [1,2], Wenxiu Wang [1,2] and Min Lin [1,2,*]

[1] National Key Laboratory of Agricultural Microbiology, Biotechnology Research Institute,
Chinese Academy of Agricultural Sciences, Beijing100081, China; guoqiannan0709@163.com (Q.G.); zhanyuhua@caas.cn (Y.Z.); zhangwei01@caas.cn (W.Z.); wangjin@caas.cn (J.W.); yanyongliang@caas.cn (Y.Y.); wenbrwang@163.com (W.W.)
[2] Key Laboratory of Agricultural Microbiome (MARA), Biotechnology Research Institute,
Chinese Academy of Agricultural Sciences, Beijing 100081, China
* Correspondence: linmin@caas.cn; Tel.: +86-10-8210-5150
† These authors contributed equally to this work.

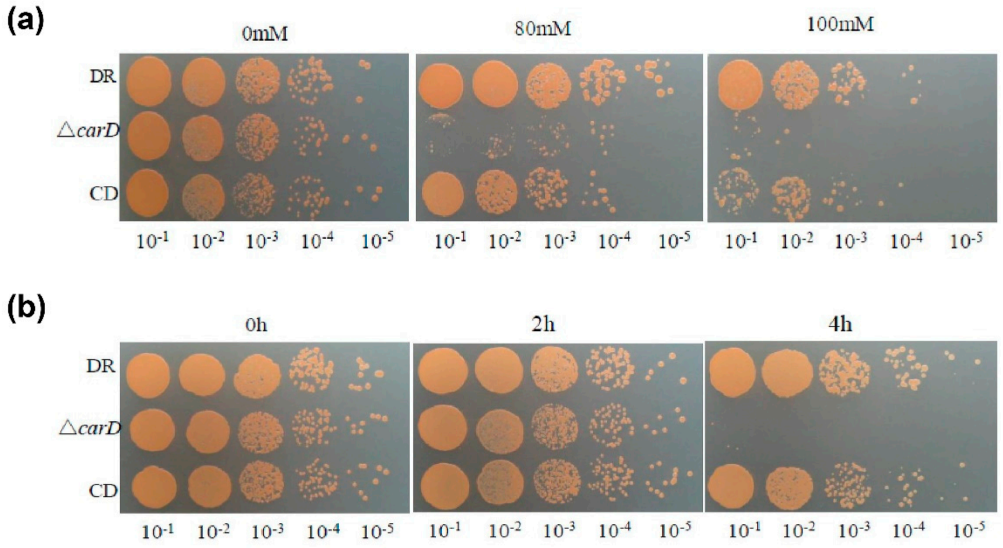

Figure. S1 Growth of the wild-type, the *carD* mutant and complementary strain (CD) upon H_2O_2 stress (a) and upon 48 °C heat stress conditions (b).

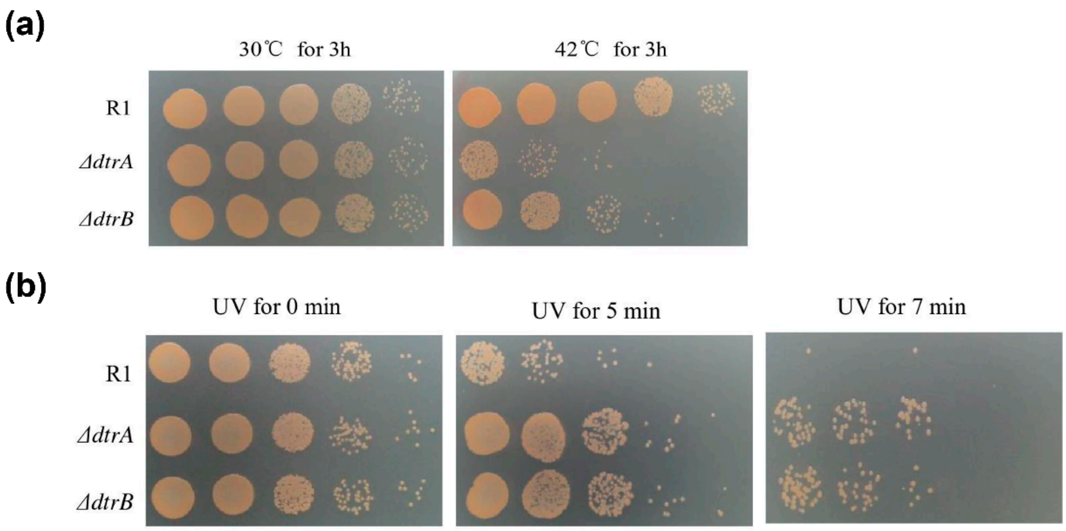

Figure. S2 Growth of the wild-type and the *dtrA/B* mutants upon 42 °C heat stress (a) and UV radiation conditions (b).

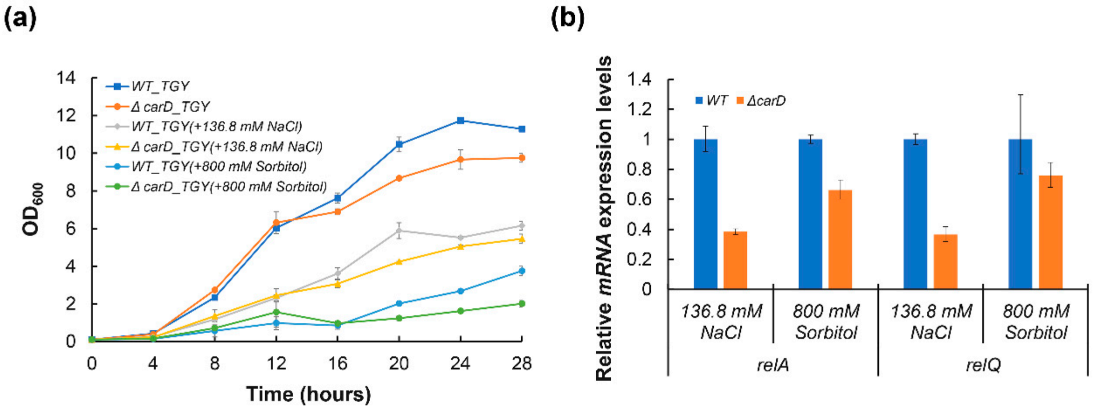

Figure. S3 Effect of *carD* gene deletion on the growth and (p)ppGpp metabolism related genes expression of *D. radiodurans* R1 under osmotic stress conditions. Growth curve of the *carD* mutant and the wild type in TGY medium, TGY medium with 136.8 mM NaCl, and TGY medium with 800 mM sorbitol (a). Expression assay of *relA* and *relQ* in WT and the *carD* mutant under osmotic stress conditions (b).

Table S1. Verification of 19 genes by quantitative real-time PCR

Locus tag	Functional description	Fold changes（T30/T0、T50/T0、T53/T0、T66/T0）	
		Real-time Q-PCR[*]	Transcriptome
DR_B0107	ribonucleotide reductase, NrdI family	8.75（±1.74）、11.93（±0.41）、10.58（±1.08）、11.98（±1.39）	7.21、4.59、4.11、4.11
DR_B0108	ribonucleotide-diphosphate reductase subunit alpha	5.76（±0.25）、7.12（±0.01）、4.92（±0.24）、5.89（±0.26）	7.11、4.47、3.48、3.61
DR_B0109	ribonucleotide-diphosphate reductase subunit beta	5.32（±0.16）、5.98（±0.02）、4.73（±0.11）、5.04（±0.30）	7.26、3.81、2.73、2.93
DR_B0121	Iron ABC transporter, ATP-binding protein	0.25（±0.06）、0.16（±0.01）、0.24（±0.02）、0.21（±0.03）	0.28、0.11、0.15、0.14
DR_B0122	iron ABC transporter permease	0.32（±0.06）、0.15（±0.04）、0.24（±0.03）、0.23（±0.01）	0.24、0.10、0.15、0.14
DR_B0123	iron ABC transporter permease	0.41（±0.01）、0.18（±0.01）、0.26（±0.02）、0.19（±0.05）	0.20、0.12、0.15、0.16
DR_1239	MaoC family dehydratase	1.15（±0.12）、1.07（±0.06）、0.38（±0.03）、0.37（±0.02）	0.88、0.91、0.39、0.30
DR_B0073	PTS fructose transporter subunit IIBC	0.12（±0.02）、0.06（±0.01）、0.08（±0.16）、0.46（±0.03）	0.38、0.07、0.03、0.23
DR_B0074	PTS fructose transporter subunit IIA	0.12（±0.01）、0.05（±0.01）、0.03（±0.01）、0.43（±0.09）	0.31、0.05、0.04、0.29
DR_1291	D-3-phosphoglycerate dehydrogenase	2.26（±0.24）、4.64（±0.09）、5.68（±0.43）、8.57（±0.01）	2.91、3.46、3.29、4.23
DR_1493	NADH dehydrogenase subunit M	5.66（±0.22）、6.27（±0.22）、10.92（±0.74）、11.59（±0.74）	6.77、6.77、13.18、8.11
DR_1495	NADH-quinone oxidoreductase subunit K	8.85（±0.96）、11.72（±0.69）、17.63（±0.78）、22.94（±0.03）	6.77、6.82、14.12、9.19
DR_1496	NADH dehydrogenase subunit J	7.65（±0.34）、10.10（±0.71）、13.69（±1.02）、18.46（±1.89）	5.03、5.50、9.85、6.23
DR_1497	NADH-quinone oxidoreductase subunit I	7.24（±0.32）、8.93（±0.70）、14.94（±1.59）、17.23（±0.98）	8.17、6.36、12.55、7.21
DR_1498	NADH-quinone oxidoreductase subunit H	7.79（±0.16）、9.46（±0.08）、18.47（±1.03）、20.85（±0.94）	7.57、6.06、12.30、7.41
DR_1778	3-isopropylmalate dehydratase large subunit 2	3.22（±0.12）、5.59（±0.36）、9.33（±0.10）、6.47（±0.24）	3.78、3.84、4.38、4.00
DR_1998	catalase	4.41（±0.19）、8.89（±0.67）、6.58（±0.11）、8.29（±0.25）	5.21、6.36、4.89、4.50
DR_1220	iron transporter	0.43（±0.09）、0.23（±0.02）、0.13（±0.02）、0.38（±0.09）	0.16、0.16、0.30、0.33
DR_1198	translational GTPase TypA	0.58（±0.01）、0.44（±0.01）、0.85（±0.05）、0.70（±0.02）	0.66、0.30、1.71、0.56

[a] Experiments were carried out from three independent RNA preparations (biological replicate) and means values with standard deviations are shown.

Table S2. Functional groups of differentially expressed genes in mutant strain Δ *drRRA* under biofilm formation condition at all stages

	Log2(Fold change)					
**Locus Tag**	**M0/T0**	**M30/T30**	**M50/T50**	**M53/T53**	**M66/T66**	**Functional description**
**Down-regulated genes**						
**T: Signal transduction mechanisms**						
DR_2418	-5.17	-5.84	-5.44	-5.17	-5.09	transcriptional regulator
DR_2363	-3.46	-3.22	-3.18	-4.24	-3.01	universal stress protein, hypothetical protein DR_2363
DR_0408	-4.27	-3.22	-2.71	-2.81	-2.63	response regulator
DR_0781	-4.79	-4.52	-3.97	-4.27	-3.96	response regulator
**C: Energy production and conversion**						
DR_A0231	-2.94	-2.47	-3.51	-4.64	-3.38	oxidoreductase
DR_A0143	-3.12	-2.39	-2.35	-2.76	-2.83	3-hydroxybutyryl-CoA dehydrogenase
DR_A0005	-4.49	-4.07	-4.87	-5.55	-4.38	alcohol dehydrogenase; zinc-containing alcohol dehydrogenase
gene3074	-5.47	-4.89	-4.50	-5.17	-4.40	cytochrome C6
**E: Amino acid transport and metabolism**						
DR_0564	-2.21	-2.63	-1.90	-2.29	-1.40	cysteine ABC transporter substrate-binding protein
DR_A0135	-3.90	-3.61	-3.70	-4.53	-3.69	ABC transporter periplasmic substrate-binding protein
DR_A0274	-4.28	-3.84	-3.86	-4.30	-3.58	flavin monoamine oxidase-like protein
**F: Nucleotide transport and metabolism**						
DR_A0235	-2.15	-3.70	-3.35	-3.46	-2.28	(2Fe-2S)-binding protein; oxidoreductase iron-sulfur subunit
DR_A0233	-4.70	-4.21	-4.19	-5.24	-4.24	aldehyde oxidoreductase; oxidoreductase iron-sulfur subunit
**G: Carbohydrate transport and metabolism**						
DR_A0047	-1.74	-2.47	-1.75	-2.01	-1.36	phosphohexose mutase
DR_A0203	-1.98	-2.80	-3.08	-3.36	-2.26	oxidoreductase
DR_0561	-2.01	-1.92	-2.06	-2.43	-2.17	sugar ABC transporter substrate-binding protein
DR_1689	-3.19	-2.68	-2.36	-2.79	-2.41	glucose-1-phosphate adenylyltransferase
DR_1790	-4.40	-4.65	-5.21	-5.17	-4.11	yellow-like protein
**I: Lipid transport and metabolism**						
DR_0166	-2.68	-3.75	-2.50	-2.69	-2.84	acyl-CoA-binding protein
**L: Replication, recombination and repair**						
DR_0132	-1.45	-1.96	-1.88	-1.66	-1.44	polyphosphate kinase
DR_0689	-2.87	-2.62	-2.54	-3.01	-1.98	uracil-DNA glycosylase

Locus Tag	Log2(Fold change) M0/T0	M30/T30	M50/T50	M53/T53	M66/T66	Functional description
**M: Cell wall/membrane/envelope biogenesis**						
DR_0646	-1.92	-1.89	-1.46	-2.05	-1.67	GTP-binding protein Era
DR_A0031	-2.92	-3.14	-2.48	-2.48	-2.33	glucose-1-phosphate thymidylyltransferase
DR_1601	-4.00	-3.18	-2.68	-3.89	-2.94	membrane protein; nodulin 21-like protein
DR_0888	-5.56	-5.20	-4.63	-5.43	-5.20	hypothetical protein DR_0888
**O: Posttranslational modification, protein turnover, chaperones**						
gene2552	-2.17	-2.20	-1.97	-2.51	-2.11	peptidyl-prolyl cis-trans isomerase
DR_1839	-3.05	-3.14	-2.98	-3.60	-3.04	peptidylprolyl isomerase
DR_2085	-3.49	-3.28	-3.36	-4.55	-4.74	glutaredoxin
DR_0944	-4.27	-4.09	-4.40	-4.97	-4.73	thioredoxin
DR_1538	-4.99	-5.50	-5.72	-5.79	-4.97	peroxiredoxin; osmotically inducible protein C
**P: Inorganic ion transport and metabolism**						
DR_A0202	-1.90	-2.48	-3.23	-3.70	-2.55	Cu/Zn family superoxide dismutase
DR_A0259	-2.19	-2.57	-3.34	-3.64	-2.80	catalase
DR_2377	-2.25	-2.02	-1.90	-2.78	-1.83	cyclase; hypothetical protein DR_2377
						superoxide dismutase [Mn]
DR_1279	-2.42	-2.11	-1.99	-2.19	-2.40	Mn family superoxide dismutase
DR_1546	-2.96	-2.87	-2.34	-3.47	-2.61	superoxide dismutase [Cu-Zn]
DR_A0006	-5.22	-4.20	-4.99	-5.87	-4.02	cyclase
DR_2263	-5.76	-5.99	-5.51	-6.15	-5.16	Dps family DNA-binding stress response protein
**S: Function unknown**						
DR_2609	-1.82	-2.00	-1.74	-2.29	-1.41	brkB protein
DR_1467	-1.99	-2.08	-1.49	-1.86	-2.04	short chain dehydrogenase
DR_0465	-2.16	-3.89	-3.03	-2.93	-2.21	hypothetical protein DR_0465
DR_1708	-2.25	-2.84	-2.68	-2.29	-1.43	hypothetical protein DR_1708
DR_2596	-2.44	-2.33	-2.36	-2.66	-1.94	metallopeptidase; hypothetical protein DR_2596
DR_1844	-3.26	-4.85	-4.26	-3.84	-3.51	acetyltransferase; hypothetical protein DR_1844
DR_0894	-3.45	-2.93	-3.19	-3.99	-2.58	hypothetical protein DR_0894
DR_2560	-3.58	-4.18	-4.93	-5.39	-4.38	hypothetical protein DR_2560
DR_0716	-3.85	-4.03	-3.55	-3.76	-3.04	hypothetical protein DR_0716
DR_1620	-3.91	-2.53	-2.88	-2.96	-1.99	ketoacyl reductase

Locus Tag	Log2(Fold change)					Functional description
	M0/T0	M30/T30	M50/T50	M53/T53	M66/T66	
DR_0392	-4.16	-3.31	-3.46	-3.83	-3.42	hypothetical protein DR_0392
DR_1385	-4.33	-4.19	-3.92	-4.17	-3.35	hypothetical protein DR_1385
DR_0613	-4.37	-4.47	-3.78	-4.65	-3.70	hypothetical protein DR_0613
DR_0614	-4.41	-3.09	-2.19	-3.73	-2.30	hypothetical protein
DR_0799	-4.48	-3.81	-3.97	-4.70	-3.28	glucose-fructose oxidoreductase
DR_1146	-5.29	-5.54	-5.26	-5.38	-4.65	general stress protein 26
DR_1181	-5.50	-4.88	-4.08	-4.88	-4.51	pyridoxamine 5'-phosphate oxidase; hypothetical protein DR_1181
DR_1199	-6.30	-6.48	-6.08	-6.68	-5.77	protease I
DR_1314	-8.71	-9.07	-9.08	-9.01	-7.78	hypothetical protein DR_1314
**No COG assignment**						
DR_1066	ND*	-5.49	-4.06	-3.45	-4.71	hypothetical protein DR_1066
DR_1317	-1.56	-2.60	-1.99	-1.83	-2.21	hypothetical protein DR_1317
DR_0818	-1.66	-2.49	-1.80	-2.23	-1.86	hypothetical protein DR_0818
gene1694	-1.74	-2.18	-2.02	-2.51	-1.46	hypothetical protein
DR_2057	-1.77	-2.55	-1.58	-2.32	-1.64	hypothetical protein DR_2057
DR_A0144	-1.77	-1.69	-2.08	-1.90	-1.64	hypothetical protein DR_A0144
DR_1803	-1.89	-2.39	-2.77	-3.21	-1.84	epoxide hydrolase; hypothetical protein DR_1803
DR_2251	-2.19	-3.03	-2.92	-2.96	-2.34	hypothetical protein DR_2251
DR_2610	-2.22	-2.18	-1.90	-2.43	-1.98	amino acid ABC transporter periplasmic amino acid-binding protein
DR_A0228	-2.33	-2.94	-1.74	-2.11	-2.41	hypothetical protein DR_A0228
DR_2407	-2.38	-2.95	-2.96	-3.60	-2.41	hypothetical protein DR_2407
DR_1165	-2.49	-1.80	-2.00	-2.27	-1.82	membrane protein;
DR_0864	-2.49	-2.27	-2.49	-3.44	-2.73	hypothetical protein DR_0864
DR_0762	-2.58	-3.45	-2.60	-2.38	-2.30	hypothetical protein DR_0762
DR_2559	-2.77	-3.99	-2.75	-4.05	-3.62	hypothetical protein DR_2559
DR_2229	-2.77	-3.26	-2.95	-3.55	-2.84	hypothetical protein DR_2229
DR_1654	-2.94	-3.00	-2.20	-2.16	-2.26	hypothetical protein DR_1654
DR_0201	-2.96	-2.17	-2.96	-3.56	-2.33	hypothetical protein DR_0201
DR_A0364	-3.47	-3.02	-3.89	-3.83	-2.82	short chain dehydrogenase/ reductase family oxidoreductase

Locus Tag	Log2(Fold change)					Functional description
	M0/T0	M30/T30	M50/T50	M53/T53	M66/T66	
DR_0863	-3.51	-2.76	-2.99	-4.27	-3.67	hypothetical protein DR_0863
DR_1054	-3.62	-4.40	-4.55	-5.18	-4.74	hypothetical protein
gene2672	-3.69	-3.76	-3.67	-4.17	-3.32	hypothetical protein
DR_0355	-3.80	-5.13	-4.04	-4.47	-4.04	hypothetical protein DR_0355
DR_0791	-3.82	-3.81	-3.53	-4.94	-3.07	chemotaxis protein CheY; chloride peroxidase
DR_A0230	-3.87	-4.10	-3.90	-3.85	-3.41	hypothetical protein DR_A0230
DR_0421	-3.94	-4.08	-2.56	-3.32	-1.87	hypothetical protein DR_0421
DR_0404	-3.94	-3.02	-3.68	-4.53	-3.99	hypothetical protein DR_0404
DR_0644	-3.95	-4.79	-4.64	-4.88	-4.27	hypothetical protein DR_0644
DR_2439	-3.96	-2.84	-2.49	-3.10	-2.86	DNA damage-inducible protein DinB;
DR_1432	-3.97	-4.75	-4.81	-4.93	-4.11	hypothetical protein DR_1432
DR_1348	-4.18	-5.74	-4.82	-4.25	-3.61	hypothetical protein DR_1348
DR_1306	-4.22	-3.98	-3.65	-4.49	-3.43	hypothetical protein DR_1306
DR_0331	-4.29	-5.03	-4.93	-6.01	-5.07	hypothetical protein DR_0331
DR_2527	-4.32	-4.48	-4.87	-5.02	-4.23	hypothetical protein DR_2527
DR_0972	-4.32	-3.88	-3.91	-4.91	-3.96	hypothetical protein DR_0972
DR_1768	-4.44	-4.49	-4.06	-4.71	-4.05	hypothetical protein
DR_0877	-4.57	-4.51	-4.83	-5.80	-5.15	hypothetical protein DR_0877
DR_1172	-4.62	-4.91	-4.45	-4.56	-4.29	hypothetical protein DR_1172
DR_1331	-4.62	-5.88	-6.22	-6.56	-5.52	hypothetical protein DR_1331
DR_1804	-4.75	-5.12	-5.70	-5.28	-4.52	cupin; hypothetical protein DR_1804
DR_0105	-4.77	-5.05	-4.57	-5.38	-3.75	hypothetical protein DR_0105
DR_1480	-4.80	-4.43	-4.69	-5.84	-4.84	AlgP-like protein
DR_1897	-4.80	-5.71	-5.52	-4.51	-3.59	hypothetical protein DR_1897
DR_1185	-4.80	-4.27	-3.85	-3.75	-2.98	S-layer-like array-like protein
DR_1176	-4.90	-5.39	-5.30	-5.42	-4.75	hypothetical protein DR_1176
DR_1962	-4.98	-4.64	-4.67	-5.84	-5.05	hypothetical protein DR_1962
DR_0746	-5.00	-4.44	-2.56	-5.14	-4.71	hypothetical protein DR_0746
gene2316	-5.00	-2.46	-4.79	-3.39	-2.91	hypothetical protein DR_2131
DR_1936	-5.01	-4.68	-5.36	-7.08	-5.81	hypothetical protein DR_1936
DR_0920	-5.18	-6.02	-5.55	-7.06	-5.72	M-like protein

Locus Tag	Log2(Fold change) M0/T0	M30/T30	M50/T50	M53/T53	M66/T66	Functional description
DR_1840	-5.19	-5.05	-5.25	-5.50	-4.55	hypothetical protein DR_1840
DR_1671	-5.42	-5.09	-4.89	-4.95	-4.84	hypothetical protein DR_1671
gene2200	-5.52	-5.85	-5.30	-6.69	-3.68	hypothetical protein
DR_1021	-5.64	-5.92	-5.15	-5.82	-4.70	hypothetical protein DR_1021
DR_2175	-5.73	-5.48	-6.08	-7.48	-6.34	hypothetical protein DR_2175
gene846	-5.74	-3.61	-2.59	-2.86	-2.37	hypothetical protein
DR_2563	-5.85	-6.27	-6.54	-7.43	-5.62	hypothetical protein DR_2563
gene287	-5.86	-3.33	-4.48	-4.22	-3.82	hypothetical protein
DR_1261	-6.25	-4.42	-4.60	-4.08	-2.89	hypothetical protein DR_1261
DR_1987	-6.28	-5.18	-5.69	-7.06	-6.05	Uncharacterized protein DR_1987
DR_2240	-6.39	-6.98	-6.96	-6.95	-5.98	hypothetical protein DR_2240
DR_1697	-6.58	-6.70	-6.06	-6.22	-5.16	hypothetical protein DR_1697
DR_1315	-6.76	-5.82	-5.90	-6.40	-5.25	hypothetical protein DR_1315
DR_1483	-7.00	-6.06	-5.74	-7.21	-5.78	hypothetical protein DR_1483
DR_2344	-7.03	-7.04	-7.60	-8.27	-6.84	hypothetical protein DR_2344
DR_1539	-7.09	-8.08	-7.33	-7.20	-7.18	hypothetical protein DR_1539
DR_1067	-7.25	-8.70	-8.43	-9.06	-8.63	hypothetical protein DR_1067
DR_0800	-7.41	-9.61	-10.19	-10.56	-7.79	hypothetical protein DR_0800
DR_2389	-8.65	-8.04	-7.87	-9.66	-8.61	transglycosylase associated protein
**Up-regulated genes**						
DR_1813	2.45	2.66	2.96	2.25	1.55	hypothetical protein DR_1813
DR_2291	2.22	2.04	1.82	2.21	1.86	cell wall glycyl-glycine endopeptidase
gene2849	2.96	1.79	1.43	1.95	1.80	bacteriophytochrome heme oxygenase
DR_0731	2.39	1.98	2.22	2.72	2.44	response regulator
rna8	ND*	ND#	-3.47	ND#	-3.14	-
gene1217	ND#	ND#	ND*	ND#	ND#	-
rna19	ND#	ND#	ND#	-1.66	-3.29	-

Genome location of some new locus tag: gene1217: CP015081.1:1172634-1172786; gene1694: CP015081.1:1648165-1648608; gene2672: CP015081.1:2609251-261032; gene2200: CP015081.1:2147018-21471; gene846: CP015081.1:805493-805681; gene287: CP015081.1:286639-286818; gene2849: CP015082.1:159622-160218.

*There is no FPKM value in corresponding biofilm formation stage sample of mutant, therefore the gene expression is downregulated.

#There is no FPKM value in corresponding biofilm formation stage sample of WT, therefore the gene expression is upregulated.

Table S3. Strains and plasmids used in this study

Strains / Plasmid	Characteristics	Sources
***Deinococcus radiodurans* R1**	Wide type	Laboratory stock (origin: CGMCC 1.633)
Δ*drRRA*	*D. radiodurans drRRA*-deletion mutant, Km^r	Laboratory construction and stock
drRRA-com	Δ*drRRA* containing the complementation plasmid pRADZ3-drRRA, Km^r, Cm^r	This study
drRRA-pRADZ3	Δ*drRRA* containing the complementation plasmid pRADZ3, Km^r, Cm^r	This study
Δ*drBON1*	*D. radiodurans drBON1*-deletion mutant, $Spec^r$	This study
Δ*irrE*	*D. radiodurans irrE*-deletion mutant, $Spec^r$	Laboratory construction and stock
Δ*dtrA*	*D. radiodurans dtrA*-deletion mutant, Km^r	Laboratory construction and stock
Δ*dtrB*	*D. radiodurans dtrB*-deletion mutant, Km^r	Laboratory construction and stock
Δ*pprM*	*D. radiodurans pprM*-deletion mutant, Km^r	Laboratory construction and stock
Δ*carD*	*D. radiodurans carD*-deletion mutant, Km^r	Laboratory construction and stock
Δ*dr_0891*	*D. radiodurans dr_0891*-deletion mutant, Km^r	Laboratory construction and stock
CD	Δ*carD* containing pRADZ3-carD, Cm^r and Km^r	Laboratory construction and stock
Escherichia coli		
Trans 10	Host for cloning vectors	TransGen
pRADZ3	Shuttle plasmid between *E. coli* and *D. radiodurans* ampicillin in *E. coli*, and chloromycetin in *D. radiodurans*	Laboratory stock
pKatAAD2	To amplify the spectinomycin resistance gene	Laboratory stock

Table S4. Primers used in this study

Primer[a]	Sequence (5′-3′)	Amplicon size, bp	Purpose
dr_0392-Up-F	CCCGAAGCGGTCGGCAGTGG	828	Δ*drBON1*(Δ*dr_0392*) construct
dr_0392-Up-R	ATGCGAGCTCGAATTCACGTCTCCTGTGGGCAGAG		
dr_0392-Sp-F	CTCTGCCCACAGGAGACGTGAATTCGAGCTCGCAT	946	
dr_0392-Sp-R	GCTCAAACTCAGTGAGAATTATTTGCCGACTACCTTG		
dr_0392-Down-F	CAAGGTAGTCGGCAAATAATTCTCACTGAGTTTGAGC	810	
dr_0392-Down-R	GCGAGCCGCACCACCAACAT		
P9 (dr_0392-ORF-F)	ATGACCCGTGACCGCAATG	1 398	Δ*dr_0392* verification
P10 (dr_0392-ORF-R)	TTACTTCTGGGTGCTGCTGC		
P11 (YZ- dr_0392-F)	GTCCTCACCTTGACCGATGG	3263 (WT)；	
P12 (YZ- dr_0392-R)	CGTGCCCACCCGCACGCTCG	2774(Δdr_0392)	
drRRA-com-F	TACCCGGGAGCTCGAATTCTAGAAGCTTCTTGATCATC GAGCGGCCTTC	1 819	*drRRA-com* construct
drRRA-com-R	GCATGCCTGCAGGTCGAATCGGATCCTCAGCCGCGCA GGGCGTAG		
qRT-16S rRNA-F	ATTCCTGGTGTAGCGGTG	146	qRT-PCR for the 16S rRNA

Primer[a]	Sequence (5′-3′)	Amplicon size, bp	Purpose
qRT-16S rRNA-R	CATCGTTTAGGGTGTGGAC		
qRT-dr_0888-F	AGTGACAGTGCTGCAACAGG	108	qRT-PCR for the dr_0888
qRT-dr_0888-R	ATCTGGACGGGAGTTGAG		
qRT-dr_0392-F	TACGGGCAGTCTCAGTCGTA	179	qRT-PCR for the dr_0392
qRT-dr_0392-R	TTCTGCACCTGCACTTCGAT		
qRT-dr_1480-F	AAGAAAGGCAACGCTCTGGT	101	qRT-PCR for the dr_1480
qRT-dr_1480-R	GTCTTGGCAGCCTTTTCGAC		
qRT-dr_1936-F	CATGCCCGAGCGTTTGC	103	qRT-PCR for the dr_1936
qRT-dr_1936-R	ATTAGAGGTCGGCTGAGGCT		
qRT-dr_0408-F	CGGGCATTCTTCCCATTCAG	143	qRT-PCR for the dr_0408
qRT-dr_0408-R	CAGCAGAAAATCGAGCGCC		
qRT-dr_1067-F	ACTGCTCAAGACCGTTGCC	140	qRT-PCR for the dr_1067
qRT-dr_1067-R	GCCAGCCCCAGTCGAAG		
qRT-dr_0561-F	CCTTGACTGTCTGGACCCAC	106	qRT-PCR for the dr_0561
qRT-dr_0561-R	ACGCTGACGATGTTGACCTT		
qRT-dr_1538-F	GGCAGATATTGCACGCAAGG	238	qRT-PCR for the dr_1538

Primer[a]	Sequence (5′-3′)	Amplicon size, bp	Purpose
qRT-dr_1538-R	GTCGAGCGCCTTGATCTCAT		
qRT-dr_2363-F	ATGACCCAGCCCGTTTCTTC	111	qRT-PCR for the dr_2363
qRT-dr_2363-R	CACTTCAAGGGCGTGCAAC		
qRT-dr_B0107-F	CGTCTACGACTCACTGACGG	132	qRT-PCR for the dr_B0107
qRT-dr_B0107-R	CCGAAGGTGTAGGTCAGCAG		
qRT-dr_B0108-F	TTCGTGGACGTGTTTTTCGC	110	qRT-PCR for the dr_B0108
qRT-dr_B0108-R	TGATAGCGGCTGCCTTCAAA		
qRT-dr_B0109-F	GGTCCGAACCCGAAGACAAT	139	qRT-PCR for the dr_B0109
qRT-dr_B0109-R	ATGTACGTCCAGCGTTCCTC		
qRT-dr_B0121-F	AAGTGACCAGCATCATCGGG	147	qRT-PCR for the dr_B0121
qRT-dr_B0121-R	GCTTCTGGGCAATGGCTCTA		
qRT-dr_B0122-F	CTGAGTGCCGGGATATTGCT	149	qRT-PCR for the dr_B0122
qRT-dr_B0122-R	AGCTCCAAGACGAGTTGCC		
qRT-dr_B0123-F	TTTACTGCTTCTCTGGCGCT	104	qRT-PCR for the dr_B0123
qRT-dr_B0123-R	GTCTGCACCACCAGACTCTC		

Primer[a]	Sequence (5′-3′)	Amplicon size, bp	Purpose
qRT-dr_1239-F	CTGGCCGCTATTTCGAGGAA	103	qRT-PCR for the dr_1239
qRT-dr_1239-R	ATGGTGAGCGTGGTGAAGAG		
qRT-dr_B0073-F	CTTTTCGATTGCCGACCGAC	121	qRT-PCR for the dr_B0073
qRT-dr_B0073-R	CACGAAGTACCCGGCGATAA		
qRT-dr_B0074-F	AGGACAACGCCGAAGTGTTC	102	qRT-PCR for the dr_B0074
qRT-dr_B0074-R	CCACGATTTTGACACCCACG		
qRT-dr_1291-F	CGTGCTGATGGGTTACCTGT	141	qRT-PCR for the dr_1291
qRT-dr_1291-R	ACGATCACCTCGGTCTGGTA		
qRT-dr_1493-F	TTTCGCACATGGGTTTCGTG	124	qRT-PCR for the dr_1493
qRT-dr_1493-R	ATCCCCACCGCCATAAACAG		
qRT-dr_1495-F	CCGACCTCGTATTACCTCGC	112	qRT-PCR for the dr_1495
qRT-dr_1495-R	TGAGCATCAGCTCCACACTC		
qRT-dr_1496-F	CGTTTGAAGCGGTGAGCATT	100	qRT-PCR for the dr_1496
qRT-dr_1496-R	GTTTCATCAGTCACGCCGTC		
qRT-dr_1497-F	CGAAATCAACATGCTGCGCT	102	qRT-PCR for the dr_1497
qRT-dr_1497-R	CGGTAATCGGCCATCTCGAA		

Primer[a]	Sequence (5′-3′)	Amplicon size, bp	Purpose
qRT-dr_1498-F	GTGGGGAGCACCAACTTTCT	127	qRT-PCR for the dr_1498
qRT-dr_1498-R	GGTTGACTTCGGCAAAGCTG		
qRT-dr_1778-F	GGAAGGGCTGCTCGAAATCT	134	qRT-PCR for the dr_1778
qRT-dr_1778-R	CGAAGTTGCGGTTGGAACTC		
qRT-dr_1998-F	ACCAACATCCAGTCGCAG	167	qRT-PCR for the dr_1998
qRT-dr_1998-R	CCTTCACCCTGGTCGTTG		
qRT-dr_1220-F	GCGGGACATATCGTTTCCCT	132	qRT-PCR for the dr_1220
qRT-dr_1220-R	CACCCGCAGCTCTATCGG		
qRT-dr_1198-F	GAACTCAAGCACGGGGAAGA	137	qRT-PCR for the dr_1198
qRT-dr_1198-R	GGGGTGTCCACGATGTTGAT		
qRT-dr_2418-F	ACCACCCCGACCTGATTTTG	144	qRT-PCR for the dr_2418
qRT-dr_2418-R	CGAGCAGCCTCACCTTTTCT		
qRT-dr_0920-F	GACTTGCAGTTCATGGGCAC	117	qRT-PCR for the dr_0920
qRT-dr_0920-R	CATCACGTCGACCTTGTCCA		
qRT-dr_1790-F	GACACCGCCGGAAACATCTA	138	qRT-PCR for the dr_1790
qRT-dr_1790-R	GTAGCTGCCCTGTATTGCCA		

Primer[a]	Sequence (5′-3′)	Amplicon size, bp	Purpose
qRT-dr_A0202-F	GAACCGCAGTCTGGTCATCC	176	qRT-PCR for the dr_A0202
qRT-dr_A0202-R	CTTTCTTCGCGTCGTAGGC		
qRT-dr_A0259-F	TGGGCAAGATGGTCCTCG	113	qRT-PCR for the dr_A0259
qRT-dr_A0259-R	CAGCGGGTCGTTGGTGA		
qRT-dr_0791-F	GAACAACCGCATGATGTCCG	100	qRT-PCR for the dr_0791
qRT-dr_0791-R	GTTCTCGATGCTCACGGTCT		
qRT-dr_0997-F	GGGCTGACCCTGCGCTATGTC	215	qRT-PCR for the dr_0997
qRT-dr_0997-R	TTGCCGACGAGGCGGTAGATG		
qRT-relA-F	CGCCTACGAGTACCTGCAAA	127	qRT-PCR for the *relA*
qRT-relA-R	CTCCACCCATTCGAGCAGTT		
qRT-relQ-F	TCAACATCCACCACGTCACC	148	qRT-PCR for the *relQ*
qRT-relQ-R	GCCACGTCCGACTCGAAATA		

[a] F, forward; R, reverse
